# Analgesic effect of percutaneously absorbed non-steroidal anti-inflammatory drugs: an experimental study in a rat acute inflammation model

**DOI:** 10.1186/1471-2474-9-15

**Published:** 2008-01-31

**Authors:** Miho Sekiguchi, Masayoshi Shirasaka, Shin-ichi Konno, Shin-ichi Kikuchi

**Affiliations:** 1 Department of Orthopaedic Surgery, Fukushima Medical University School of Medicine, 1-Hikarigaoka, Fukushima City, Fukushima 960-1295, Japan; 2 Department of Pharmacy, Fukushima Medical University Hospital, 1-Hikarigaoka, Fukushima City, Fukushima 960-1295, Japan

## Abstract

**Background:**

External medication that is absorbed percutaneously may be used to reduce inflammation and relieve pain from acute injuries such as ankle sprains and bruises. The plaster method of percutaneous absorption for non-steroidal anti-inflammatory drugs (NSAIDs) was established in Japan in 1988. However, due to the possibility of a placebo effect, the efficacy of this method remains unclear. This experimental study was conducted to control for the placebo effect and to study the efficacy of the plaster method in relieving pain by using a rat model of inflammation.

**Methods:**

Male Wistar-Imamichi rats were used. A yeast suspension was injected into the right hind paw to induce inflammation. A sheet (2.0 × 1.75 cm) containing the drug was adhered to the inflamed paw. Five treatment groups were used, and each sheet contained a single drug: loxoprofen sodium (loxoprofen-Na) (2.5 mg); felbinac (1.75 mg); indomethacin (1.75 mg); ketoprofen (0.75 mg); or base only (control, 0 mg). Mechanical pain threshold, expression of c-Fos in the dorsal horn, and amount of prostaglandin (PG) E_2 _in the inflamed paw were evaluated.

**Results:**

Pain threshold increased after treatment, and was significantly increased in the loxoprofen-Na group compared with the control group (*p *< 0.05). Amounts of PGE_2 _were significantly decreased in the loxoprofen-Na and indomethacin groups compared with the control group (*p *< 0.05). Expression of c-Fos was significantly decreased in the loxoprofen-Na group compared with the control group (*p *< 0.05).

**Conclusion:**

Percutaneously absorbed NSAIDs have an analgesic effect, inhibit expression of c-Fos in the dorsal horn, and reduce PGE_2 _in inflamed tissue, indicating the efficacy of this method of administration for acute inflammation and localized pain.

## Background

Non-steroidal anti-inflammatory drugs (NSAIDs) are commonly used in clinical situations worldwide to reduce inflammation and pain. These agents are administered orally, intravenously, intrarectally and percutaneously. Topical applications for percutaneous absorption comprise several forms, such as ointments, lotions, aerosols, liniments, cataplasms and plasters, and pressure-sensitive adhesives. A plaster method of percutaneous absorption for NSAIDs was developed in Japan in 1988. This application is used to reduce inflammation and to relieve pain from acute injuries such as sprains and bruises. This differs from patch-type drugs for systemic administration. However, in clinical situations, external medicines are not typically used as a main conservative treatment because the efficacy of this method in humans remains unclear due to the possibility of a placebo effect. Since the effects of plaster treatment can be separated from the placebo effect, an experimental study was needed to evaluate the efficacy of this method. Few preclinical experiments have investigated the analgesic effects of percutaneous absorption with these types of drugs.

NSAIDs inhibit the biosynthesis of prostaglandins (PGs), and PGE_2 _is one of the inflammatory mediators associated with inducing peripheral hyperalgesia [1]. PGE_2 _levels can be used to determine the effect of the medication as an index of inflammation. In addition, the *c-Fos *gene is expressed following noxious input, and level of expression offers a marker of signals to sensory cells in the spinal cord [2-4]. The purpose of this study was to investigate the effects of plaster treatment on mechanical hyperalgesia, expression of c-Fos in the spinal cord, and the amount of PGE_2 _in a rat model of inflammation.

## Methods

A total of 90 male Wistar-Imamichi rats (4–5 weeks old; Imamichi Institute of Animal Reproduction, Japan) were used in this study. Animals were housed in plastic cages at room temperature with a 12:12 light:dark cycle and *ad libitum *access to food and water. All experiments were approved by the Animal Studies Committee at Fukushima Medical University.

### Acute inflammatory model

**Figure 1:**
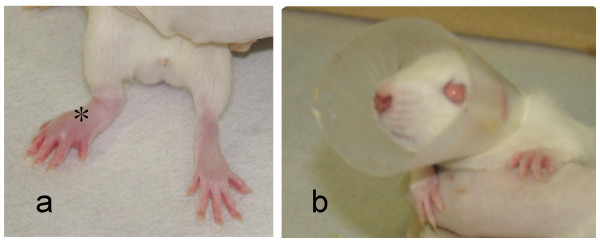
**Acute inflammatory model and plastic Elizabethan collar for the rat neck**. a) A suspension of yeast was injected intradermally into the right paw (*). b) A plastic Elizabethan collar was fixed around the neck of each rat to prevent ingestion of the drug sheet.

Animals were anesthetized using 99% diethyl ether (Wako Pure Chemical Industries, Osaka, Japan). A suspension of 10% or 20% brewer's yeast (Sigma-Aldrich, MO, USA) at 0.1 ml/hind paw was injected intradermally into the right hind paw. According to Randall and Selitto [5], 20% brewer's yeast induces inflammation. Pain threshold is reduced at 1 h and is further decreased at 2 and 4 h. A return to baseline is seen by 48 h after induction of inflammation. However, efficacy of treatment with use of 20% yeast was only found according to PGE_2 _level, and not pain threshold or c-Fos expression (data not shown). As the degree of inflammation using 20% yeast thus appeared too strong to evaluate pain threshold and c-Fos expression by the percutaneous drug delivery system, 10% yeast was used. To prevent ingestion of the drug sheet, an Elizabethan collar comprising a plastic sheet was applied around the neck of each rat (Fig. 1).

### Treatment groups

**Figure 2:**
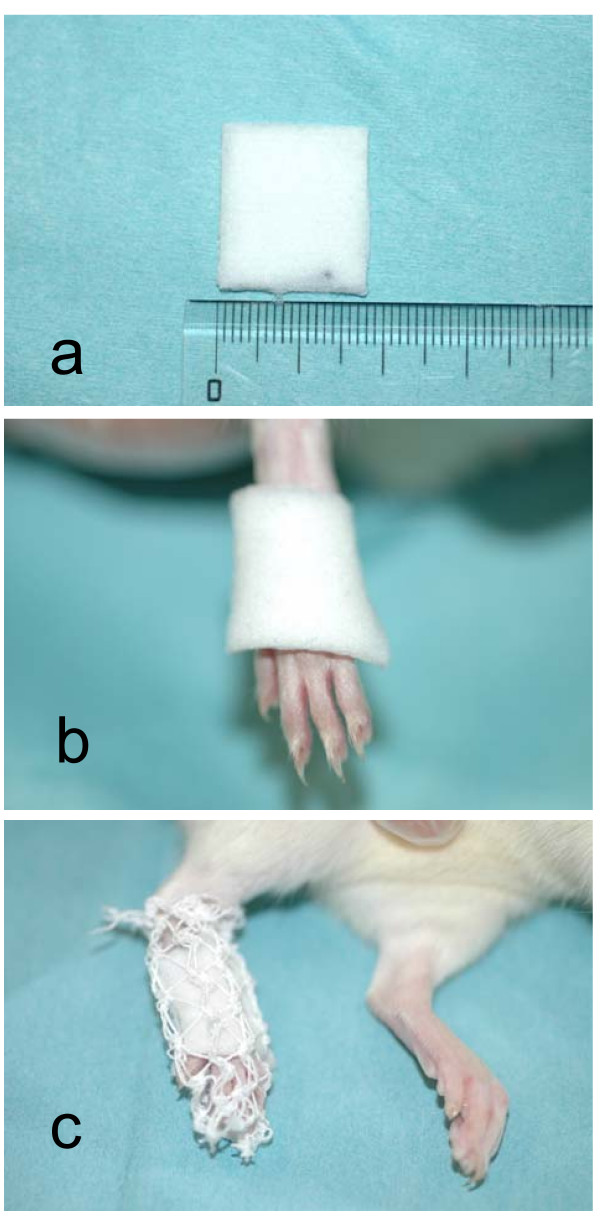
**Sheet application**. a) Sheet size was 2.0 cm × 1.75 cm. b) The sheet was applied to the paw. c) The foot was covered by a net to prevent the sheet peeling off.

Animals were divided into 5 groups and treated with different percutaneously absorbed drugs. All drugs were clinically applied in the form of a patch sheet (10.0 × 14.0 cm) to the affected area. Drug dose per sheet was 100 mg loxoprofen sodium (loxoprofen-Na), 70 mg felbinac, 70 mg indomethacin or 30 mg ketoprofen. Since the density of each drug imbedded in the sheet differed, the applied dose was controlled by standardizing sheet size in this study at 2.0 cm × 1.75 cm, based on the relative difference between human and rat body sizes. The sheet for rat treatment contained 2.5 mg loxoprofen-Na, 1.75 mg felbinac, 1.75 mg indomethacin or 0.75 mg ketoprofen. Control rats received an application of a base sheet, representing a sheet without NSAIDs (control; 0 mg). This base sheet contained several substances to improve drug absorption, control drug release and minimize skin irritation. To exclude the influence of such substances, treatment groups were compared to the control group treated with the base sheet alone. A sheet of each drug was applied to the paw for treatment and covered by a net to prevent the sheet from coming off (Fig. 2).

### Measurement of pain threshold

Pain threshold was measured using 30 rats (n = 6 per group). Treatments were started 4.5 h after 10% yeast injection. The pain threshold began to decrease at 1 h and decreased further by 2 and 4 h, returning to baseline by 48 h after induction of inflammation [5]. In addition, swelling of the paw is reportedly maximized at 2–5 h after inflammation is induced and is maintained for more than 5 h [6]. As a result, to investigate the efficacy of NSAID sheets, treatments were started on the basis of active inflammation according to the clinical situation, and pain thresholds were measured. Experiments were performed according to the method of Randall and Selitto [5], with the same modifications used by Winter and Flataker [6]. Just before treatment, pain thresholds were measured on bilateral paws. Pain thresholds were measured using the Analgesy-Meter (Ugo Basile, Comerio, Italy), which is able to gradually apply pressure to the paw, and the degree of pressure is shown on a graduated scale. Struggling or vocalization was considered as a positive pain response. Two sheets were adhered to the dorsal and plantar sides of paw. Pain thresholds were measured 3 h after treatment to compare groups. Measurement of pain thresholds was performed once on each rat at each time point. Data are shown as mean ± standard deviation. Statistical analysis was performed using the Bonferroni/Dunn test. Values of *p *< 0.05 were considered statistically significant.

### Amount of PGE_2 _in inflamed tissue

Levels of PGE_2 _were assayed using 35 rats (n = 6 in each treatment group; n = 5 for treatment-naïve rats). Induced inflammation was not administered to naïve rats, and they were not given any treatment. Paws were treated with treated or control sheets immediately after injection of 20% yeast suspension. One sheet was adhered to the dorsal side of the paw.

The amount of PGE_2 _was measured 0.5 h after treatment. Since PGE_2 _induces peripheral hyperalgesia [1], production of PGE_2 _might start shortly after injection. To determine whether drugs inhibit PGE_2 _production, drugs sheets were adhered immediately after injection. In addition, if drugs inhibit production of PGE_2_, expression of c-Fos would be related to this response, so we chose the same period for these measurements. Rats were euthanized using 99% diethyl ether, then inflamed paws were isolated 0.5 h after treatment, immediately frozen with liquid nitrogen and stored at -80°C. Whole paws were crushed in a Cryo-press (Microtec, Tokyo, Japan), then homogenized with a Polytron PT-3100 homogenizer (Kinematica, NJ, USA) in ice-cold phosphate-buffered saline (PBS) supplemented with 10 mM EDTA and 100 μM indomethacin (Sigma-Aldrich). The homogenate was centrifuged, and the supernatant fraction was stored at -20°C until the PGE_2 _assay. PGE_2 _content was assayed using a PGE_2 _EIA kit (Cayman Chemical Company, MI, USA). All raw data are shown in the graph. Statistical analyses were performed using the Bonferroni/Dunn test. Values of *p *< 0.05 were considered statistically significant.

### Expression of c-Fos in the dorsal horn

Histological findings were analyzed using 25 rats (n = 5 per group). Paws were treated with treated or control sheets immediately after 10% yeast suspension injection. Two sheets were adhered on the dorsal and plantar sides of the paw. Perfusion was conducted 0.5 h after treatment. Expression of c-Fos is known to peak at 30 min in the superficial laminae of the dorsal horn after nociceptive stimulation [7]. We therefore investigated expression of c-Fos 30 min after stimulation and treatment. After perfusion with 200 ml of 4% paraformaldehyde-0.1 M PBS, the spinal cord at the L5 level was removed. This section of spinal cord was then immersed in 4% paraformaldehyde-0.1 M PBS solution for 1 h, in a 10% sucrose-PBS solution for 24 h, and in a 20% sucrose-PBS solution for 24 h. A 40-μm frozen section was made from the spinal cord using a microtome. Sections were collected as floating sections in 0.1 M PBS, and these were immersed in 0.1 M PBS including 0.2% Triton X for 3 days. Sections were immersed in 1% blocking serum for 30–60 min, then reacted with anti-c-Fos antibody (1:3000; Santa Cruz Biotechnology, CA, USA) at a temperature of 4°C for 48 h. Sections were then reacted using the avidin-biotin complex method (Vector Laboratories, CA, USA) for 30 min, followed by washing in biotinylated IgG antibody (Vector Laboratories) for 30 min. Afterwards, sections were stained using a 3,3'-diaminobenzidine, 0.0045% hydrogen peroxide solution and placed on a glass slide to keep dry.

A microscope connected to a computer was used at 400× magnification. The c-Fos-immunoreactive neurons were observed and counted on the computer monitor with a KS 100 imaging system (Carl Zeiss, Hallbergmoos, Germany). After confirmation of layers in the spinal dorsal horn in accordance with reports by Molander et al. [8], images at 400× magnification were imported into the computer to check for c-Fos-immunoreactive neurons. From 30 sections per sample test rat, 5 sections were chosen with the maximum number of c-Fos-immunoreactive neurons for layers I–VI. The spinal dorsal horn was divided into 3 groups: layers I–II; III–IV; and V–VI. The mean number of c-Fos-immunoreactive neurons for layers I–II was set as a measured value for a test rat. Statistical analysis was performed using a Bonferroni/Dunn test. Values of *p *< 0.05 were considered statistically significant.

## Results

### Measurement of pain threshold

**Figure 3:**
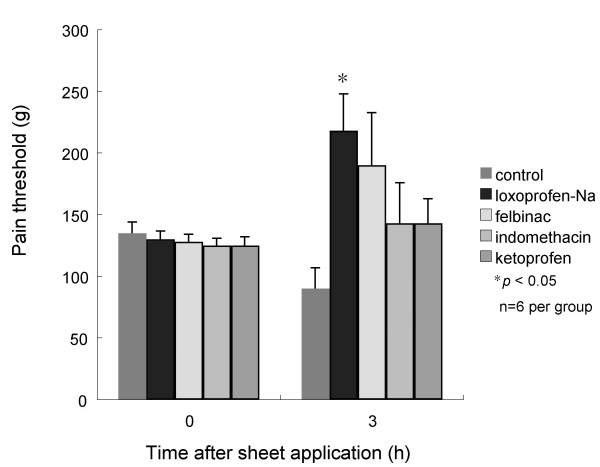
**Pain threshold in inflamed paws (n = 6 per group)**. No difference was seen among groups at 4.5 h after injection of yeast suspension (0 h). Pain threshold increased 3 h after treatment in treated groups. In the loxoprofen-Na group, pain threshold significantly increased compared with the control group (*p < 0.05).

Pain threshold of normal paws (contralateral side) was 255.0 ± 17.0 g, compared to 136.0 ± 4.0 g for inflamed paws 4.5 h after injection (0 h after treatment). Pain threshold in the control group decreased to 90 ± 17 g, while that in the other 4 groups was increased 3 h after treatment (Fig. 3). In the loxoprofen-Na group, pain threshold was significantly increased compared with the control group (*p *< 0.05).

### Amount of PGE_2_

**Figure 4:**
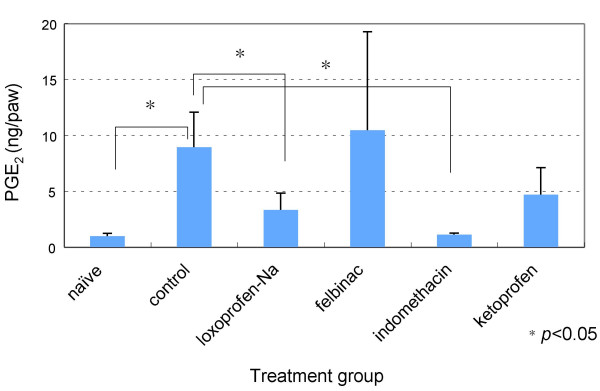
**PGE_2 _in inflamed paws**. In the control group, the amount of PGE_2 _increased 0.5 h after inducing inflammation. A significant difference in the amount of PGE_2 _was identified between the control group and treatment-naïve animals (*p < 0.05). The amount of PGE_2 _in the groups treated with loxoprofen-Na and indomethacin was decreased compared with the control group (*p < 0.05) (n = 5 for naïve group; n = 6 for treatment groups).

Amount of PGE_2 _was 1.01 ± 0.25 ng in treatment-naïve rats, and 8.97 ± 3.1 ng in the control group. Amounts of PGE_2 _were 3.34 ± 1.53 ng in the loxoprofen-Na group, 10.49 ± 8.78 ng in the felbinac group, 1.16 ± 0.13 ng in the indomethacin group and 4.72 ± 2.42 ng in the ketoprofen group. Significant differences in amounts of PGE_2 _were seen between control group and the loxoprofen-Na and indomethacin groups (*p *< 0.05) (Fig. 4).

### Expression of c-Fos

**Figure 5:**
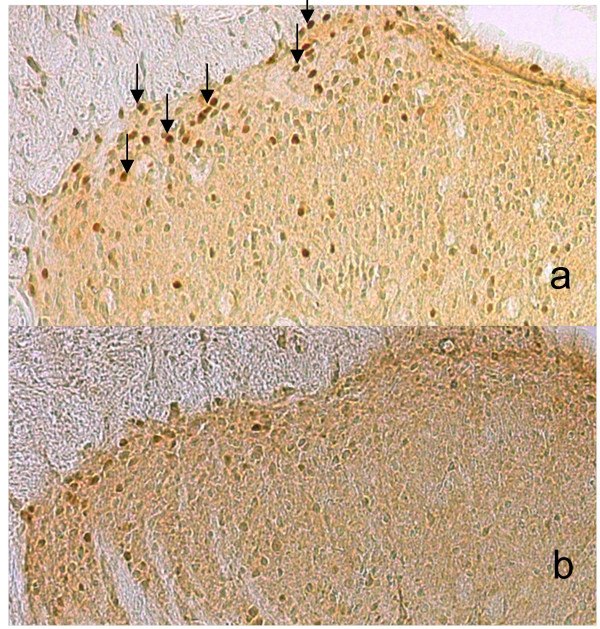
**Immunohistochemical findings of c-Fos-immunoreactive cells in the dorsal horn ipsilateral to the inflamed hind paw (n = 5 per group)**. Neurons displaying c-Fos immunoreactivity (arrows; brown staining) in the superficial layer (I–II) for control (a) and loxoprofen-Na-treated (b) groups.

Immunoreactivity to c-Fos was observed in superficial laminae of the dorsal horn in all groups (Fig. 5). Numbers of c-Fos-immunoreactive cells in the 4 treatment groups were decreased compared with the control group (Table 1). In particular, c-Fos-immunoreactivity was significantly less in the loxoprofen-Na group than in the control group (*p *< 0.05) (Table 1).

**Table 1:** Number of c-Fos-immunoreactive cells in superficial laminae of the ipsilateral dorsal horn (n = 5 per group)


	Control	Loxoprofen-Na	Felbinac	Indomethacin	Ketoprofen

Mean ± SEM	134.8 ± 4.5	113.7 ± 4.7	125.3 ± 5.4	127.5 ± 4	127.2 ± 3.5
p value vs. control		0.0008*	0.1268	0.2164	0.2227

The number of c-Fos-immunoreactive cells was decreased in the loxoprofen-Na group compared with the control group (* *p* < 0.05).

## Discussion

NSAIDs are the most widely used drugs for reduction of inflammation and pain in clinical situations [9]. However, side effects upon oral administration of NSAIDs include gastrointestinal disturbance and hepatic dysfunction. Reducing these side effects while maintaining the drug's therapeutic effects is important, as is exposing the target region to the drugs for a suitable length of time. To reduce side effects and enhance therapeutic effects, changing the route or method of administration is effective even without changing the chemical structure. Drugs can be given orally, intravenously, intrarectally or percutaneously. Blood concentrations of NSAIDs rise after oral administration, and systemic side effects may result. In contrast, percutaneous absorption only acts locally and is expected to increase drug concentrations and produce higher effects at the site of inflammation, thus reducing side effects throughout the whole body. Percutaneously delivered NSAIDs were thus developed to reduce inflammation and pain. Percutaneous absorption allows drugs to permeate the skin and affect the local area [10]. However, this method is not used as main clinical treatment due to the placebo effect. Depending on the substances facilitating permeability of NSAIDs through the skin, patients feel coldness or warmth on the skin and can also notice various smells. These factors can act to create placebo effects, influencing assessment of the effect of plasters that contain NSAIDs. The present experimental study was performed to exclude such possible placebo effects.

Changes in the diameter of the paw in this acute inflammatory model have been reported in experimental studies after oral administration of NSAIDs, but not following percutaneous absorption [11]. However, no reports have described analgesic effects after topical administration of NSAIDs in an experimental study. In this study, percutaneous absorption of NSAIDs displayed analgesic effects compared with the control group. Loxoprofen sodium was particularly effective. Although inflammation had already developed before treatment, percutaneously absorbed NSAIDs were effective in increasing pain thresholds locally. This result suggests that the local concentration of NSAIDs in inflamed tissue increases following permeation through the skin and is sufficient to achieve analgesic effects.

Analgesic effects of NSAIDs are associated with inhibition of PG production. In particular, PGE_2 _and PGI_2 _increase the effect of bradykinin, a pain-inducing substance. PGE_2_, one of the principal inflammatory mediators, reportedly contributes to the induction of peripheral hyperalgesia and allodynia [1]. In the present study, yeast-induced inflammation increased PGE_2 _content in the paw. This increase of PGE_2 _may contribute to local inflammation and decreased pain thresholds. The amount of PGE_2 _decreased after treatment, so percutaneous absorption of NSAIDs may have inhibited PGE_2 _production to reduce pain in the inflamed hind paw. Treatment was started just after inducing inflammation, and the time interval between treatment and PGE_2 _measurement was 30 min. Percutaneously absorbed NSAIDs thus appear to work quickly. PGE_2 _levels at later post-treatment time points were not measured, which was a limitation to this study. However, inhibition of PG biosynthesis is known to indirectly inhibit bradykinin generation. Since increases in pain threshold remained present 3 h after initiation of treatment in this study, PGE_2 _biosynthesis was assumed to be inhibited even after 30 min.

Expression of the c-Fos gene is an effective way to signal sensory cells in a spinal cord and trigeminal nuclei excited by noxious inputs. In particular, c-Fos-activated cells are found in the ipsilateral superficial layer (I and II) in the dorsal horn of the spinal cord 2 h after noxious input such as mustard oil, formalin and carrageenin [2-4,12,13]. A previous study identified c-fos mRNA in the spinal cord after the formalin test, and expression of c-Fos peaked at 30 min in the superficial laminae of the dorsal horn (laminae I–II) and at 1–3 h in the deep laminae (laminae V–VI) [7]. Expression of c-Fos mRNA in those regions appears to correspond to early- and late-phase responses to the formalin test [14]. Some reports have evaluated NSAID effects for oral and intravenous administration using c-Fos expression in the spinal cord [9,14-21]. However, no previous studies have investigated the expression of c-Fos after treatment by percutaneously absorbed NSAIDs. In the present study, percutaneous local absorption of NSAIDs into inflamed tissue inhibited expression of c-Fos in the spinal cord compared with controls. Loxoprofen-Na was especially effective in decreasing c-Fos expression. These results suggest that loxoprofen-Na can be effective in the early stage of inflammation induction compared with other drugs. Treatment for >25 min prior to induction of inflammation has been performed to evaluate expression of c-Fos [11,19-21]. In this study, treatment was started just after inducing inflammation, and only one treatment group could prevent c-Fos expression. However, other time points and durations of effect were not investigated and thus this may be limitations to this study. In addition, dose and drug metabolism of NSAIDs differ depending on whether treatment is oral or by percutaneous absorption, so the effect of percutaneously absorbed NSAIDs cannot be directly compared with oral NSAIDs. Nevertheless, NSAIDs administered systemically affect not only inflamed tissues, but also tissues where prostanoids play physiological roles. This means that oral administration of NSAIDs carries risks of adverse effects such as gastric ulcer and edema. In contrast, percutaneously absorbed NSAIDs affect only the local area, so the risk and severity of adverse effects might be reduced compared with oral NSAIDs.

We investigated the effect of percutaneously absorbed NSAIDs by measuring pain threshold, amount of PGE_2 _and expression of c-Fos in the dorsal horn. No previous reports have used these three categories to investigate the effects of percutaneously absorbed NSAIDs. We found that these effects are not placebo effects, and percutaneous absorption of NSAIDs can be expected to be useful as a main method of conservative treatment instead of oral administration. In particular, percutaneous absorption of NSAIDs is likely to be useful for patients with localized disease or risk factors, and for elderly individuals with a higher risk of side effects.

## Conclusion

Percutaneously absorbed NSAIDs have analgesic effects, inhibit expression of c-Fos in the dorsal horn, and reduce PGE_2 _levels in inflamed tissues. This type of topical application may be useful for acute inflammation and localized pain.

## Competing interests

The author(s) declare that they have no competing interests.

## Authors' contributions

All authors participated in the design of the study. MSE performed the studies and drafted the manuscript. MSH and SKO performed statistical analyses. SKO and SKI participated in coordination and helped to draft the manuscript. All authors have read and approved the final version of the manuscript.

## Pre-publication history

The pre-publication history for this paper can be accessed here:


